# Evaluating the potential impact of interruptions to neglected tropical disease programmes due to COVID-19

**DOI:** 10.1093/trstmh/trab023

**Published:** 2021-03-06

**Authors:** T Déirdre Hollingsworth, Pauline Mwinzi, Andreia Vasconcelos, Sake J de Vlas

**Affiliations:** Big Data Institute, Li Ka Shing Centre for Health Information and Discovery, University of Oxford, Old Road Campus, Headington, Oxford OX3 7LF, UK; Expanded Special Project for Elimination of Neglected Tropical Diseases (ESPEN), World Health Organization Regional Office for Africa, P.O.BOX 06 Cité du Djoué Brazzaville, Congo; Big Data Institute, Li Ka Shing Centre for Health Information and Discovery, University of Oxford, Old Road Campus, Headington, Oxford OX3 7LF, UK; Department of Public Health, Erasmus MC, University Medical Center Rotterdam, P.O. Box 2040, 3000 CA Rotterdam, the Netherlands

**Keywords:** neglected tropical diseases, coronavirus, modelling

At the time of writing (February 2021), it is 1 y since the coronavirus disease 2019 (COVID-19) pandemic began to become a pandemic. To date, there have been >100 million cases of COVID-19 diagnosed globally, leading to at least 2.2 million deaths.^1^ The indirect effects of the pandemic on other health conditions, as well as economic and societal well-being, are accruing at an alarming rate.^2^ Probably due to its young population, perhaps combined with recent experience of combating large-scale epidemics, such as Ebola in 2014 and 2016, sub-Saharan Africa has been affected relatively modestly by COVID-19, with a death toll of about 60 000 cases so far, mainly in South Africa. However, these reported numbers may be underestimates, given the limitations of the local health systems. Also, the future course of the pandemic is highly uncertain, especially where large-scale vaccination campaigns may be more challenging than in high-income countries. Model predictions suggest that suspension of control efforts for malaria, TB and HIV in low- and middle-income countries could lead to deaths on a similar scale to those from COVID-19 itself.^3^ Similar effects were seen during the Ebola outbreak in West Africa, where the number of excess deaths due to other factors (including maternal mortality and particularly malaria) was estimated to be of the same order of magnitude as the number of deaths directly due to Ebola.^4^

## COVID-19 and neglected tropical diseases

Only recently, on 28 January 2021, the WHO celebrated the global achievements in striving towards the 2020 goals for neglected tropical diseases (NTDs) and announced the roadmap to achieve further goals in 2030.^5^ This celebration of achievements combined with a future commitment to lessen the toll of these diseases were overshadowed by the potential impact of COVID-19 on health systems in populations where NTDs are endemic. In April 2020, the WHO issued a recommendation to postpone mass drug administration (MDA) and other control activities for NTDs until further notice, in recognition of the potential risks of these activities to increase COVID-19 infection.^6^ In July 2020, the WHO provided a decision-making framework, including a risk-benefit assessment, for resuming or maintaining activities in the context of COVID-19 (guidance on the risk analysis).^7^ Many NTD-related activities have ceased and the picture of programme interruptions is still uncertain.

As part of broad stakeholder engagement,^8^ the NTD Modelling Consortium was asked by national programmes, donors, policymakers and implementation partners to estimate the consequences of interrupting NTD interventions and the impacts of mitigation strategies, especially regarding attempts to achieve the 2030 roadmap targets, building on previous work in this area.^9^ Initial results were presented on seven NTDs, namely, the Gambiense form of human African trypanosomiasis (gHAT) and visceral leishmaniasis in the Indian sub-continent, which are both controlled by case finding, as well as lymphatic filariasis, onchocerciasis, schistosomiasis, soil-transmitted helminthiases and trachoma, which are controlled by preventive chemotherapy through MDA.^7^^–^^11^ The results were also presented at WHO webinars (https://www.who.int/news/item/26-05-2020-neglected-tropical-diseases-and-covid-19-who-holds-consultative-meeting-to-assess-impact-on-programme-implementation, https://apps.who.int/iris/bitsteam/handle/10665/339238/sea-cd-328-eng.pdf?sequence=1) and an extensive slide-deck was circulated to partners.^12^ This special issue of *Transactions of the Royal Society of Tropical Medicine and Hygiene* reports the more detailed findings of the modelling studies for each of the seven NTDs following further stakeholder engagement.

During any interruption to a NTD programme, there is likely to be a resurgence in infection and/or disease to levels above those which would have been observed if the programme had been maintained. The extent and consequences of this additional transmission depends on the dynamics of each individual NTD. For the seven NTDs studied in detail here, we outline some of the potential drivers of high impact.

### Epidemic growth rate

The dynamics of these seven NTDs are quite different and this has important consequences for the resurgence of infection. Onchocerciasis^13^ and lymphatic filariasis^14^ generally have very slow epidemic growth rates, and so the resurgence of infection during one missed round of annual MDA is generally very slight. Basically, the delay in reaching the target is similar to the number of years that treatment rounds are missed. The dynamics of gHAT^15^ are also slow, meaning that short-term interruptions to case finding may have limited impact. By contrast, visceral leishmaniasis in the Indian subcontinent,^16^ as well as trachoma^17^^,^^18^ and schistosomiasis,^19^ have somewhat faster epidemic growth rates, particularly in high transmission areas. For visceral leishmaniasis in the Indian subcontinent, as well as trachoma and schistosomiasis, interruptions to programmes will therefore undermine the gains made over many years and considerably delay achievement of the 2030 targets. For soil-transmitted helminths^20^ there are a range of resurgence rates, approximately in proportion to the inverse of the worm’s lifespan.^21^

### Accrual of morbidity

Although most of the models in this collection do not explicitly consider morbidity, it is clear that the rate at which morbidity accrues for a given increase in prevalence is also an important consideration in prioritising the restarting of programmes. For schistosomiasis, visceral leishmaniasis and trachoma, particularly in high transmission settings, the impact on morbidity or even mortality may be substantial.^16–19^

### Prevalence prior to interventions

For any NTD, the areas at highest risk of resurgence during interruptions to programmes are those with high transmission rates, or high prevalence before interventions were introduced. As resurgence is more rapid, the longer the delay then the longer the time it will take to get back on track (e.g. a larger number of MDA rounds). The analyses in this collection also highlight that the impact of an interruption early or late in a programme may have different effects. For schistosomiasis and onchocerciasis, the model predictions clearly show that programmes with shorter MDA histories will be most vulnerable to interruptions, as the underlying transmission potential is highest.^13^^,^^19^ The same can be seen for visceral leishmaniasis in the Indian subcontinent in terms of the impact on cumulative visceral leishmaniasis incidence. However, if the impact is viewed in terms of the delay in reaching the 2030 target (i.e. incidence below 1 visceral leishmaniasis case per 10 000 people per year), then the impact is highest later in the programme (5–8 y), because the already achieved target will be lost again.^16^

### Extended delays to programmes and affected additional interventions

The longer the interruption to programmes then the greater the potential negative impact. The analyses presented in this collection demonstrate that these are non-linear effects, especially for NTDs with a high epidemic growth rate, and the second year may have a higher impact than the first year of delay depending on the epidemic growth rate and the prevalence at the time of interruption. Similarly, for NTDs that benefit from additional interventions, such as vector control (for onchocerciasis) or bednets (for lymphatic filariasis), interruption of these interventions will further increase the overall impact.^13^^,^^14^

### Coverage and efficacy when programmes return

COVID-19 has had diverse impacts on programmes and some commentators suggest^2^^,^^22–24^ that there may be issues regarding coverage for MDAs when programmes are implemented in 2021 and beyond. For most of the simulations in this collection, the results contain an assumption that programmes will return with the same coverage and efficacy as was in place before 2020. If this is not achieved then mitigation of the impact of COVID-19 on NTD programmes will be extremely challenging.

### Mitigation strategies

Ideally, interruptions of NTD control due to the COVID-19 pandemic should be minimised and programmes should start again as soon as possible. Mitigation strategies will help all NTD programmes to recover the lost ground, but in different ways. In the analyses within this issue, additional or enhanced MDA programmes were simulated for the five diseases for which MDA is the major intervention. For yearly MDA programmes, this included providing the 2020 round in 2021, switching to biannual treatment from 2021, including adults in school-based programmes (for soil-transmitted helminthiases and schistosomiasis) or only targeting children (for trachoma). These mitigation strategies have important logistical and economic costs, and the expected coverage in these alternative scenarios should be carefully considered. However, their inclusion in these analyses provides an epidemiological investigation of their potential to mitigate the impact of COVID-19-related delays.

Clearly, increasing the number or coverage of MDAs reduces the level of infection more rapidly, but there were some interesting results. For onchocerciasis, for example, biannual MDA turned out to be a better choice for mitigation than increasing coverage.^13^ For soil-transmitted helminthiases, mitigation may not be strictly necessary to achieve the 2030 targets, as the programmes will catch up before this date. However, the duration until catching up will be shortened, thereby reducing the impact on morbidity. For schistosomiasis, increasing coverage and extending the programmes by treating adults will both help to achieve the targets. In particular, for schistosomiasis and trachoma, the authors propose that maintaining mitigation strategies will be the only way to achieve 2030 targets in high-endemicity settings, as the recommended strategy would not have managed this.^25^^,^^26^ In this way, the possible mitigation strategies would not only allow programmes to catch up to where they would have been, but also to accelerate achieving the 2030 goals.

For the case-finding disease (in this special issue that includes visceral leishmaniasis and sleeping sickness [or gHAT]), interruptions to either passive or active case detection is likely to lead to a build-up of undetected cases that will need to be addressed when programmes return to full strength, and these cases may take some time to be addressed and for transmission to reduce again.^15^^,^^16^

## Modelling and policy

The studies in this special issue have benefitted from the models that have been developed by the NTD Modelling Consortium over the 6 y of its existence; some of them even have a history of many more years to decades.^27^ For gHAT, onchocerciasis and trachoma, the calculations were based on two (or for lymphatic filariasis, three) different models per NTD capturing the same basic mechanisms of transmission, natural history and control, but using different underlying assumptions and methodologies. The fact that multiple models largely agreed regarding the predicted impact of interruptions and mitigation strategies supports the general outcome of the different papers. 

The studies further benefitted from the partnerships and discussions that underlie the modelling decisions on the right scenarios and outputs to study. The global NTD community has supported this work through both the WHO and the Expanded Special Project for Elimination of NTDs (ESPEN),^8^ as well as by national programme managers engaging with the early publication of the report. This is the first special issue produced by the NTD Modelling Consortium that explicitly accounts for the extent to which its five principles for policy-relevant modelling have been followed.^28^ This was achieved by providing the policy-relevant items for reporting models in epidemiology of neglected tropical diseases (PRIME-NTD) Table in the supplements of each of the papers. The PRIME-NTD Table contains a checklist of how each principle was satisfied and where this can be found in the manuscript. The first and perhaps most important principle (i.e. engaging stakeholders throughout) was only met to a limited extent, given the short time span in which to deliver the outcomes of these studies. However, mainly existing models were used, and most of them, especially those with a long history, have had involvement of various stakeholders at different stages of model development and extension. The next three principles (i.e. a complete description of models, the data used and degree of uncertainty) are basic components of good modelling practice and are dealt with by detailed supplements, including uncertainty and sensitivity analyses. The fifth and final principle (i.e. to provide testable model outcomes) is extremely well satisfied by the many presented trends of how the number of infections may change as a consequence of COVID-19-induced interruptions of control and the chosen mitigation strategies. In the coming years these trends can be compared with data to help in validating and further improving the models.

## Looking forword

Over the last year there has been broad discussion that this may be an opportunity for innovation in response to the crisis, and this collection contributes to this discussion by highlighting that practical steps are needed to deliver this potential,^29^ that novel methods for data collection can optimise the information underlying decisions^30^ and, importantly, that novel strategies can deliver not just mitigation but acceleration of programmes.^31^ Importantly, as the modelling analyses in this special issue demonstrate, NTD programmes need to be functioning at the same, or at an even higher level than previously, to mitigate the impact of this devastating pandemic on NTDs.

## Data Availability

None.
